# IsoMiRmap: fast, deterministic and exhaustive mining of isomiRs from short RNA-seq datasets

**DOI:** 10.1093/bioinformatics/btab016

**Published:** 2021-01-20

**Authors:** Phillipe Loher, Nestoras Karathanasis, Eric Londin, Paul F. Bray, Venetia Pliatsika, Aristeidis G. Telonis, Isidore Rigoutsos

**Affiliations:** Computational Medicine Center, Sidney Kimmel Medical College, Thomas Jefferson University, Philadelphia, PA 19107, USA; Computational Medicine Center, Sidney Kimmel Medical College, Thomas Jefferson University, Philadelphia, PA 19107, USA; Computational Medicine Center, Sidney Kimmel Medical College, Thomas Jefferson University, Philadelphia, PA 19107, USA; Department of Internal Medicine, University of Utah, Salt Lake City, UT 84112, USA; Computational Medicine Center, Sidney Kimmel Medical College, Thomas Jefferson University, Philadelphia, PA 19107, USA; Computational Medicine Center, Sidney Kimmel Medical College, Thomas Jefferson University, Philadelphia, PA 19107, USA; Department of Human Genetics, Miller School of Medicine, University of Miami, Miami, FL 33136, USA; Computational Medicine Center, Sidney Kimmel Medical College, Thomas Jefferson University, Philadelphia, PA 19107, USA

## Abstract

**Motivation:**

MicroRNA (miRNA) precursor arms give rise to multiple isoforms simultaneously called ‘isomiRs.’ IsomiRs from the same arm typically differ by a few nucleotides at either their 5′ or 3′ termini or both. In humans, the identities and abundances of isomiRs depend on a person’s sex and genetic ancestry as well as on tissue type, tissue state and disease type/subtype. Moreover, nearly half of the time the most abundant isomiR differs from the miRNA sequence found in public databases. Accurate mining of isomiRs from deep sequencing data is thus important.

**Results:**

We developed isoMiRmap, a fast, standalone, user-friendly mining tool that identifies and quantifies all isomiRs by directly processing short RNA-seq datasets. IsoMiRmap is a portable ‘plug-and-play’ tool, requires minimal setup, has modest computing and storage requirements, and can process an RNA-seq dataset with 50 million reads in just a few minutes on an average laptop. IsoMiRmap deterministically and exhaustively reports all isomiRs in a given deep sequencing dataset and quantifies them accurately (no double-counting). IsoMiRmap comprehensively reports *all* miRNA precursor locations from which an isomiR may be transcribed, tags as ‘ambiguous’ isomiRs whose sequences exist both inside and outside of the space of known miRNA sequences and reports the public identifiers of common single-nucleotide polymorphisms and documented somatic mutations that may be present in an isomiR. IsoMiRmap also identifies isomiRs with 3’ non-templated post-transcriptional additions. Compared to similar tools, isoMiRmap is the fastest, reports more *bona fide* isomiRs, and provides the most comprehensive information related to an isomiR’s transcriptional origin.

**Availability and implementation:**

The codes for isoMiRmap are freely available at https://cm.jefferson.edu/isoMiRmap/ and https://github.com/TJU-CMC-Org/isoMiRmap/. IsomiR profiles for the datasets of the *1000 Genomes Project*, spanning five population groups, and *The Cancer Genome Atlas* (TCGA), spanning 33 cancer studies, are also available at https://cm.jefferson.edu/isoMiRmap/.

**Supplementary information:**

Supplementary data are available at *Bioinformatics* online.

## 1 Introduction

MicroRNAs (miRNAs) are among the best studied non-coding RNAs in the last 25 years. MiRNAs are now known to be linked to virtually all cellular processes, in homeostasis and disease, in animals and plants (Calin and Croce, 2006a, b; Lim, 2003; Reinhart, 2002).

According to the model of miRNA biogenesis that prevailed for the first nearly 20 years (Bartel, 2004, 2009), a miRNA precursor is processed to give rise to a single mature miRNA from either its left or its right arm. This sequence has been reported by miRBase (Kozomara and Griffiths-Jones, 2014) as the miRNA ‘product’ of the corresponding precursor miRNA. In what follows, we call this sequence the ‘reference’ miRNA. For nearly twenty years, the sequence of the reference miRNA served as the basis for designing quantification assays and synthetic RNAs to be used in experimental work.

**Fig. 1.**
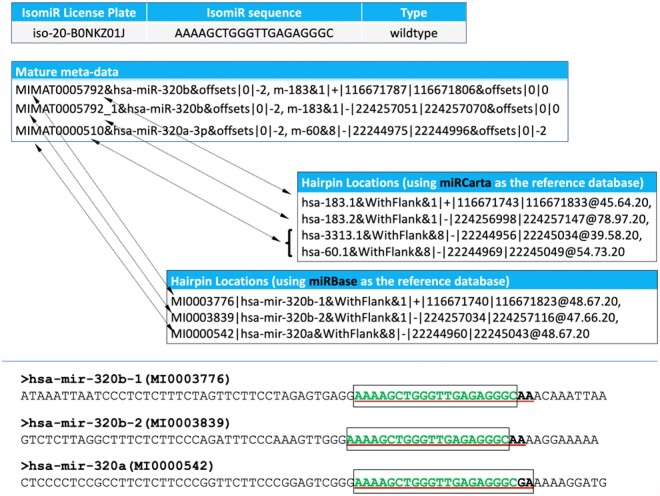
An example of isoMiRmap’s HTML output. Shown is the information for iso-20-B0NKZ01J. This isomiR can potentially arise from multiple hairpins at three different genomic locations. For two of the three mature miRNA references that correspond to these three locations, the 3′ termini differ between miRCarta and miRBase. The mature miRNA meta-data and the bottom panel illustrate these differences. For the bottom panel; the isomiR sequence is shown in green, the miRCarta reference sequence is highlighted with a box border, and the miRBase reference sequence is boldfaced and underlined

Our understanding of miRNAs evolved very quickly following the introduction of deep sequencing technologies. We now know that a miRNA precursor gives rise to a ‘cloud of isoforms’ that co-exist and are known as *isomiRs* (Rigoutsos *et al.*, 2019). For a number of years already, we have been reporting that these isomiRs show more diversity in their 3′ termini compared to their 5′ termini (Loher *et al.*, 2014; Londin *et al.*, 2020; Magee *et al.*, 2018; Telonis *et al.*, 2015c). In addition, as our large scale analyses of TCGA (Telonis *et al.*, 2017) revealed, the relative production of 5′ isomiRs versus 3′ isomiRs from a given miRNA precursor depends on context. One characteristic such example is miR-9: as can be seen in Figure 1 A-B of (Telonis *et al.*, 2017), miR-9 produces a rich set of 5′ isomiRs in low grade glioma but not in other cancer types. Our TCGA analyses also showed that the identities and abundances of isomiRs have additional dependencies that include tissue type, tissue state and cancer type/subtype (Londin *et al.*, 2020; Magee *et al.*, 2018; Telonis *et al.*, 2015c, 2017).

Unexpectedly, we found that in healthy individuals and patients the identities and abundances of isomiRs are further modulated by personal attributes that include sex, and genetic ancestry (Loher *et al.*, 2014; Magee *et al.*, 2018; Telonis *et al.*, 2015c, 2017). More recently, we linked isomiRs to overall survival (Londin *et al.*, 2020) and to the molecular events that underlie health disparities (Magee *et al.*, 2018; Telonis *et al.*, 2015c; Telonis and Rigoutsos, 2018). Clearly, these previously documented dependencies on tissue state, disease type and personal attributes make isomiRs ideal candidates for developing personalized diagnostics and therapeutics.

Given the increasing research interest in isomiRs, several tools have been proposed in recent years for the purpose of identifying them in deep-sequencing data (Baras *et al.*, 2015; Chu *et al.*, 2016; Kanke *et al.*, 2016; Liu *et al.*, 2019; Pantano *et al.*, 2010; Sablok *et al.*, 2013). In addition, databases that catalogue isomiRs and permit over-the-web queries also became available (Fehlmann *et al.*, 2017; Yang *et al.*, 2019b). These approaches rely on mapping deep-sequencing data to collections of annotated parental sequences such as miRNA precursors, transfer RNA (tRNA), ribosomal RNA (rRNA), etc. As we documented previously, mapping to collections of annotated sequences instead of the whole genome can give rise to erroneous results. For example, the genomes of higher organisms are riddled with incomplete copies of many tRNAs and rRNAs (Cherlin *et al.*, 2020; Telonis *et al.*, 2014, 2015a), which complicate the identification of the location from which the corresponding fragments arise. To identify tRNA-derived fragments (tRFs) or rRNA-derived fragments (rRFs) in deep sequencing data while avoiding the generation of erroneous results, the mapping scheme must explicitly consider the sequence idiosyncrasies of each RNA type and the distribution of its members across the genome, as well as account for the numerous incomplete instances of these RNAs (Loher *et al.*, 2017; Magee and Rigoutsos, 2020; Telonis *et al.*, 2015a,b). Analogous considerations apply to the case of miRNAs and isomiRs and are discussed in more detail below. An additional complication results from the fact that the mapping step uses general purpose mapping tools [e.g. Bowtie (Langmead *et al.*, 2009)]. Unless care is taken to modify the default parameter settings, these tools will permit insertions, deletions and replacements. Such permissive choices will generally add to the problems that arise from mapping to small annotated sets because they confound the true genomic origin of short RNAs and complicate their quantification.

In what follows, we present isoMiRmap, a new tool for identifying isomiRs *directly* from deep-sequencing data. Our design goals for isoMiRmap included several requirements: speed; self-containment; user-friendliness; accessibility to users with limited or no bioinformatics expertise; deterministic and exhaustive mapping; ability to identify 3′ non-templated post-transcriptional additions (Kim *et al.*, 2019; Song *et al.*, 2015); and, the ability to identify isomiRs containing common polymorphisms and documented mutations. Importantly, we sought to provide these capabilities while keeping the needed computational resources to a minimum so that the tool could easily be run on an average laptop. Below, in addition to describing how isoMiRmap addresses these requirements, we also compare it with several other tools using both real and synthetic data.

Before proceeding, it is important to stress that in the literature the concept of a miRNA *isoform* has been tightly coupled to that of the reference miRNA. If it so happened that two different repositories listed different sequences for the mature miRNA product of a given miRNA precursor, what would be an *isomiR* for one repository could be the *reference miRNA* for the other repository. We discuss an example of this below. Historically, the most abundant isoform from a given miRNA arm was taken to be the reference miRNA for that arm. However, as we showed previously, the identity of the most abundant isomiR differs in the general case from the sequence found in the public miRNA database, and depends on tissue type and disease type, as well as on personal attributes such as sex, and genetic ancestry (Loher *et al.*, 2014; Londin *et al.*, 2015, 2020; Magee *et al.*, 2018; Telonis *et al.* 2015c, 2017; Telonis and Rigoutsos, 2018). Since the definition of a reference miRNA is database-dependent, it is important that users be aware of this point to avoid misinterpretation. Alternatively, users can rely on using license plates (discussed below) to refer to an isomiR.

## 2 Materials and methods

### 2.1 Definitions

We define ‘miRNA-space’ as the union of all miRNA precursors that are listed in the user-selectable ‘miRNA reference set.’ An isomiR is tagged as ‘exclusive’ if its sequence is found only within miRNA-space. If its sequence is found anywhere in the reference human genome outside of miRNA space, the isomiR is tagged as ‘ambiguous.’ IsoMiRmap provides users with a choice between reference sets miRCarta Rel. 1.1 (Backes *et al.*, 2018) and miRBase Rel. 22 (Kozomara and Griffiths-Jones, 2014). IsoMiRmap uses miRCarta as the default reference set because it incorporates miRBase as well as newly discovered miRNAs (Backes *et al.*, 2016; Londin *et al.*, 2015). The miRNA-space comprises (i) ‘wild-type’ isomiRs and (ii) ‘modified’ isomiRs. The former exists within miRNA-space and their sequence matches that of the reference human genome. The latter includes isomiRs that incorporate a known polymorphism (Karczewski *et al.*, 2020; Sherry, 2001), or 3′ non-templated post-transcriptional additions (Kim *et al.*, 2019).

### 2.2 Naming and labeling notations

IsoMiRmap uses the notation that we originally introduced in 2014 (Loher *et al.*, 2014) and have been using ever since (Londin *et al.*, 2020; Magee *et al.*, 2017, 2018; Telonis *et al.*, 2015c, 2017; Telonis and Rigoutsos, 2018). The notation has already been adopted by the recently proposed mirGFF3 format standard (Desvignes *et al.*, 2020). The notation makes it easy to state how an isomiR’s endpoints differ from the annotated ‘reference’ mature found in public databases. For example, the label ‘hsa-miR-142-5p|-2|-3’ builds on the miRBase notation and refers to the isoform of miR-142-5p whose 5′-end begins 2 nucleotides (nts) upstream of the miRBase reference’s 5′-end and 3′-end terminates 3 nts upstream of the miRBase reference’s 3′-end.

In addition, our tool assigns universally unique identifiers, the “license plates,” to each isomiR. These identifiers are derived from the isomiR’s sequence using a public, previously described mapping scheme (Pliatsika *et al.*, 2016). Each isomiR has a unique ‘license plate’ and each license plate corresponds to a unique isomiR. For example, for the isomiR hsa-miR-142-5p|-2|-3 with a length of 20 nts and sequence CCCATAAAGTAGAAAGCACT, the license plate is iso-20-KQB3FBPI. Importantly, the isomiR license plate scheme does not require a brokering mechanism for issuing names and persists over time because it is does not depend on a genome assembly. Readers who wish to leverage this scheme and encode into or decode from license plates can do so programmatically by visiting https://cm.jefferson.edu/license-plates-download/, or interactively by visiting https://cm.jefferson.edu/LicensePlates/.

To accommodate legacy labels, the output of isoMiRmap also includes a *genome-assembly-dependent* naming scheme for isomiRs. This scheme allows one to quickly know the hairpin to which an isomiR belongs and preserves genomic coordinates. One key benefit of this scheme it that it allows us to report isomiRs that are expressed from previously un-annotated arms of known miRNAs while permitting the user to immediately know the identity of the parental precursor. For example, ‘hsa-144-69.1&WithFlank&17|-|58331240|58331311@7.26.20’ refers to an isomiR from the miRNA locus hsa-144-69.1 of miRCarta. The infix ‘WithFlank’ indicates that the miRCarta precursor has been padded on both the 5′ and 3′ end with flanking regions (default: 6 nts at each end) in order to allow the reporting of previously uncharacterized isomiRs. The augmented miRNA precursor is between locations 58331240 and 58331311 inclusive on the reverse strand of chromosome 17. The suffix ‘7.26.20’ indicates that the isomiR spans positions 7 through 26 inclusive of the expanded precursor and that it is 20 nts long.

### 2.3 Data acquisition and preparation

We downloaded the sequenced reads for the four short RNA normal tissue samples (TCGA-H6-8124-11A, TCGA-H6-A45N-11A, TCGA-HV-A5A3-11A and TCGA-YB-A89D-11A) of TCGA’s pancreatic ductal adenocarcinoma study (Cancer Genome Atlas Research Network. Electronic address and Cancer Genome Atlas Research, 2017) from NCI’s Genomic Data Commons Data Portal (https://portal.gdc.cancer.gov/). These reads were already pre-processed for quality and adaptor trimming. The sequenced reads for the short RNA LCL samples were downloaded from the Geuvadis RNA sequencing project (Lappalainen *et al.*, 2013). For this collection, the cutadapt tool (Martin, 2011) was used for quality trimming and removing adaptors from the reads.

### 2.4 The k-mer lookup table

IsoMiRmap makes use of a ‘k-mer lookup table.’ IsoMiRmap uses this table to achieve its fast mapping speed while still being exhaustive during the isomiR mining step. Each entry of this table is a candidate isomiR sequence comprising k nucleotides, with the value of k ranging between 18 and 26 nts. Of all possible k-mer strings, only those that can be derived from a precursor miRNA are included in this table. Since the table depends on the miRNA reference set, we provide one table for miRCarta (default) and one specifically for miRBase. We build the k-mer table in an off-line process by enumerating all possible sequence segments with lengths between 18 and 26 nts that can be found in the expanded miRNA precursors of the miRNA reference set. Each of these k-mers is then sought in the entire human genome—the current implementation and associated tables are based on the human genome assembly GRCh38—using a deterministic, exhaustive brute force search. For each k-mer, the table indicates whether it occurs in miRNA space only (‘exclusive,’ or ‘Y’), or can also be found in other regions of the genome (‘ambiguous,’ or ‘N’). Due to the layout of the k-mer table, it can be easily amended to include newly identified isomiRs that are not represented in the provided tables. In Supplementary Material, we include a detailed description of how to build this attribute table.

**Table 1.**
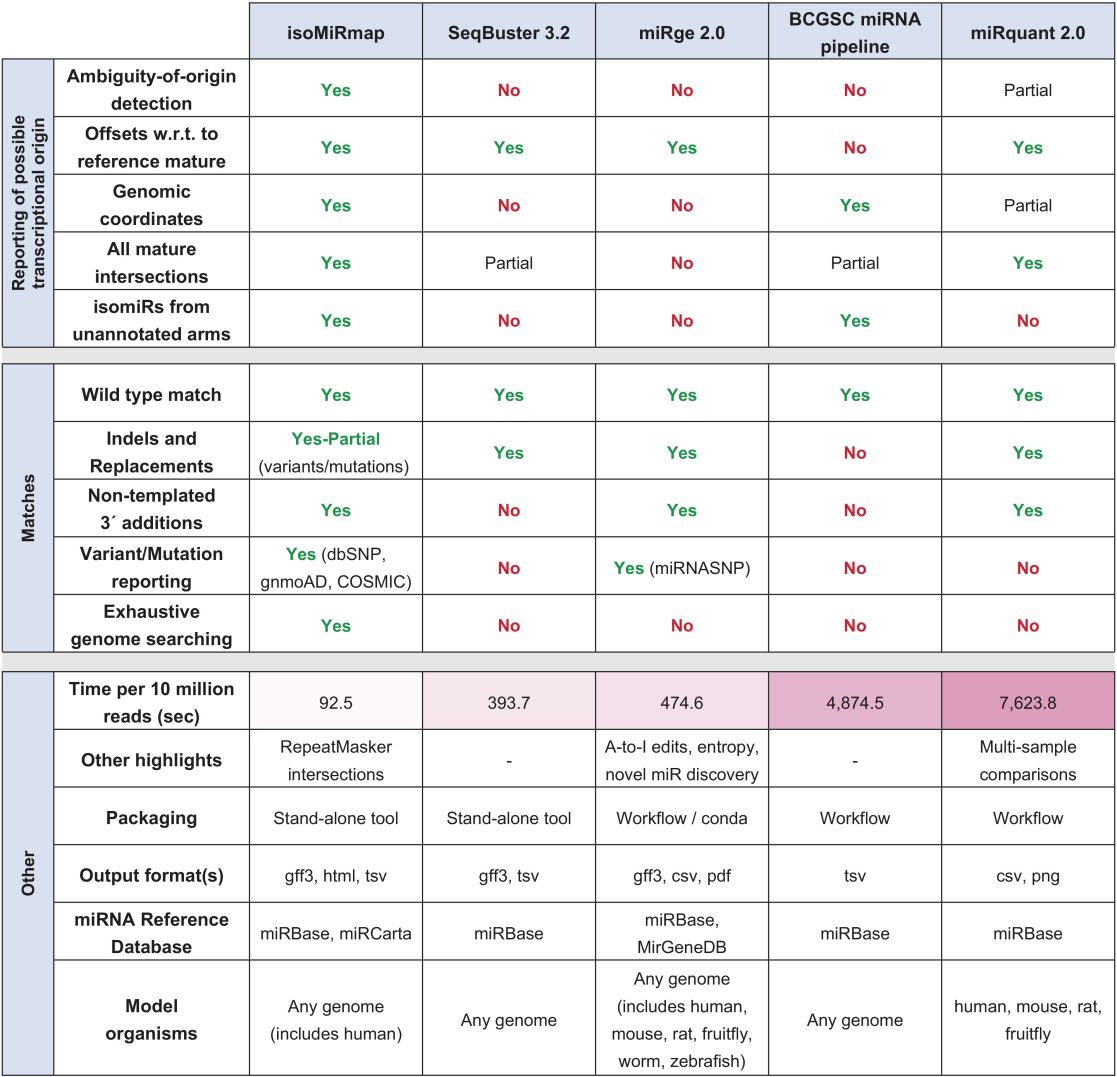
Comparative study of five different isomiR profiling approaches

*Note*: The reported timings were obtained by running each tool on a single Intel Xeon 750 processor core (2.27 GHz).

### 2.5 Attributes of k-mers: repeats

For each isomiR, isoMiRmap also reports whether the isomiR can be found in a known repeat element. To do so, an attribute table is created that lists the repeat family or families containing the isomiR sequence. IsoMiRmap recognizes the 20 classes of RepeatMasker (http://www.repeatmasker.org): SINE, SINE?, LINE, RC, RC?, RNA, scRNA, srpRNA, rRNA, tRNA, DNA, DNA?, snRNA, Retroposon, Unknown, LTR, LTR?, Satellite, Low_complexity and Simple_repeat. In Supplementary Material, we include a detailed description of how to build this attribute table.

### 2.6 Accommodating variant-containing isomiRs with a second k-mer lookup table

IsoMiRmap allows the mining of isomiRs that may contain instances of common genetic variants that are listed in either dbSNP Rel. 151 (Sherry, 2001) or the Genome Aggregation Database (gnomAD) (Karczewski *et al.*, 2020), or in the Catalog of Somatic Mutations in Cancer (COSMIC) v87 (Forbes *et al.*, 2017). IsoMiRmap considers only variants that have a minor allele frequency of ≥1% in at least one of the 26 reference populations represented in the 1000 Genomes Project (Genomes Project Consortium *et al.*, 2015). Only single nucleotide replacements, and insertions or deletions of one nucleotide are considered. Information about the polymorphism found in an isomiR is reported in the generated output. The genomic variants that are identified and reported by isoMiRmap are not detected *de novo*. Instead, they are precomputed and made part of the lookup tables that accompany the executables. For this step, we enumerate publicly known SNPs and mutations and precompute the resulting sequences. Additional variants can be added through the creation of a new lookup table as described in Supplementary Material.

### 2.7 Tool implementation

IsoMiRmap was developed as a Python application and requires Python version 3.5 or greater. Briefly, the tool operates in three phases: initialization; scanning; and, output. During the initialization phase, isoMiRmap loads into memory the different k-mer lookup tables, the attribute table, precursor sequences and the coordinates of the reference miRNAs for the miRNA database that the user selected (miRCarta or miRBase). During the same phase, isoMiRmap also inputs all sequenced reads into a frequency table to conserve memory. During the scanning phase, isoMiRmap pairs all unique sequenced reads with their corresponding entries in the k-mer and attribute tables and builds the annotations. The sequenced reads are also located within the precursor sequences in order to determine their offsets in the corresponding miRNA precursor. If a read is not found in the k-mer lookup tables, special processing that we have put in place will trim and rescan the read in order to identify possible instances of non-templated isomiRs with 3′ post-transcriptional additions (e.g. uridylation, etc.). Lastly, during the output phase isoMiRmap aggregates the information, calculates normalized expression values (in reads-per-million) and reports the isomiR profiles in three different formats: tab delimited text, HTML and miRGFF3 (Desvignes *et al.*, 2020).

## 3 Results

When presented with deep-sequenced reads from any short-RNA dataset that have been quality-trimmed and from which the adaptors have been removed, isoMiRmap traverses the reads and accesses the accompanying tables to retrieve the information for each read in turn and generates the output for the user.

### 3.1 Exhaustive reporting of parental precursors

IsoMiRmap reports the identity of all miRNA precursors from the user selected reference set, miRBase or miRCarta, that contain a given isomiR and the location within each precursor where the isomiR can be found. Figure 1 illustrates this using the 20-nt long isomiR iso-20-B0NKZ01J with sequence AAAAGCTGGGTTGAGAGGGC as an example. Using miRCarta as the reference set, isoMiRmap reports this isomiR as belonging to four different annotated hairpins from three different genomic loci, two on chromosome 1 and one on chromosome 8. This isomiR happens to appear in two precursors that overlap with one another, hsa-3313.1 and hsa-60.1 and isoMiRmap reports both of them.

### 3.2 Exhaustive reporting of alternative labels

Separately for all parental precursors of a reported isomiR, isoMiRmap also reports the isomiR’s overlap with the mature isoform, the miRNA ‘reference,’ which is listed in a reference database such as miRBase or miRCarta. We note that miRCarta and miRBase use different labeling schemes and frequently report slightly different miRNA precursor and mature sequences for the same genomic locus. This can pose complications. The practical consequence is that a given mature miRNA will appear with different labels in miRCarta and miRBase but will be assigned the same *unique* license plate by isoMiRmap. For example, the 20-nt isomiR AAAAGCTGGGTTGAGAGGGC (Fig. 1) matches exactly the endpoints of the reference mature isoforms of miRCarta’s precursors hsa-183.1 and hsa-183.2. However, the same exact sequence does not match the reference isoforms for the corresponding two miRNAs in miRBase, hsa-mir-320b-1 and hsa-mir-320b-2. In fact, this miRCarta reference mature miRNA is shorter than miRBase’s reference mature miRNA and corresponds to the latter’s 0|-2 isoform (Fig. 1).

It is important to stress that this seeming disparity is expected. As we already mentioned above, the concept of a ‘reference’ miRNA is a function of the miRNA database being used. IsoMiRmap addresses this complication by reporting a given isomiR’s identifiers for each of three collections in turn: miRCarta (Backes *et al.*, 2018), miRBase (Kozomara *et al.*, 2019), and the novel, primate-specific miRNA that we reported previously (Londin *et al.*, 2015). By erring on the side of being verbose, isoMiRmap helps the user avoid problems in downstream analyses. In addition, isoMiRmap reports the isomiR’s sequence-based *license plate* (Pliatsika *et al.*, 2016, 2018) identifier. Because it is sequence-based, this unique identifier is persistent and will remain unchanged over time and unaffected by future changes in the assembled genome, or in the labeling scheme of miRBase and miRCarta.

### 3.3 Auto-tagging isomiRs with ambiguous genomic origin

It is possible that an isomiR may not be exclusive to miRNA-space (see Section 2). We refer to such an isomiR as ‘ambiguous’ because its sequence is found both within and outside of miRNA-space. IsoMiRmap tags all wild-type isomiRs and those with 3′-end additions as either ambiguous or exclusive. By doing so, we alert the user to the possibility that the isoform may arise elsewhere on the genome and not be transcribed from an annotated hairpin.

**Fig. 2.**
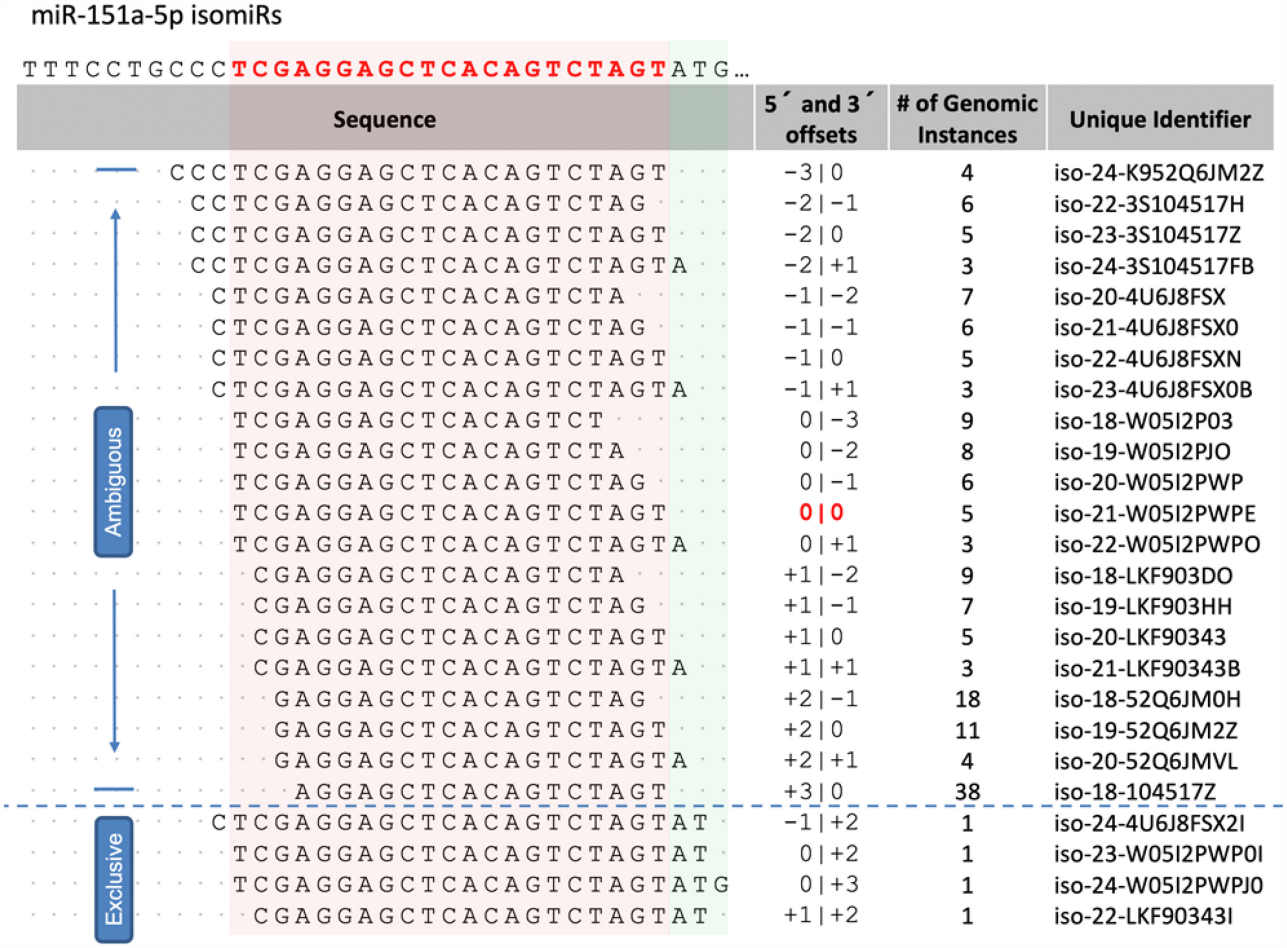
This figure shows all possible isomiRs of miR-151a-5p within 3 nts of the endpoints of the miRBase reference miRNA. The latter (0|0) is shown in red color. Note how only the isomiRs that extend at least 2 nts past the 3′ end of the reference are marked as exclusive. The remaining isomiRs exist in at least one genomic location *outside* of miRNA-space. The light red background is added to permit easy comparison of the isomiRs to the span of the miRBase mature miRNA reference. The light green background is meant to draw attention to the fact that adding two or more nucleotides to the 3′ of the miRNA reference suffices to disambiguate the genomic origin of the corresponding isomiR, whereas adding nucleotides to its 5′ end fails to do so

Figure 2 illustrates this point using miRBase’s miR-151a-5p|0|0, whose license plate is iso-21-W05I2PWPE. This isomiR maps to miRCarta’s precursor hsa-75-30.1, which is located on chromosome 8. For this miRNA locus, both miRBase and miRCarta report the same mature miRNA reference sequence. IsoMiRmap marks this isomiR as ambiguous because its sequence (TCGAGGAGCTCACAGTCTAGT) is also present at non-miRNA related regions. Specifically, the sequence exists identically on chromosome 1 (on the forward strand between locations 98892897 and 98892917, and antisense to PLPPR5), chromosome 19 (on the forward strand between locations 43460872 and 43460892, and antisense to LYPD3), chromosome X (on the reverse strand between locations 53381683 and 53381703, and within an intron of SMC1A) and chromosome 4 (on the reverse strand between locations 17447061 and 17447081, in an un-annotated region).

Figure 2 helps also illustrate another point: shorter isomiRs are expected to have more instances across the genome than longer ones and, thus, are more likely to be ambiguous. This is something that we encountered previously in our work with tRNA-derived fragments (Telonis *et al.*, 2016) whose lengths match those of isomiRs.

**Fig. 3.**
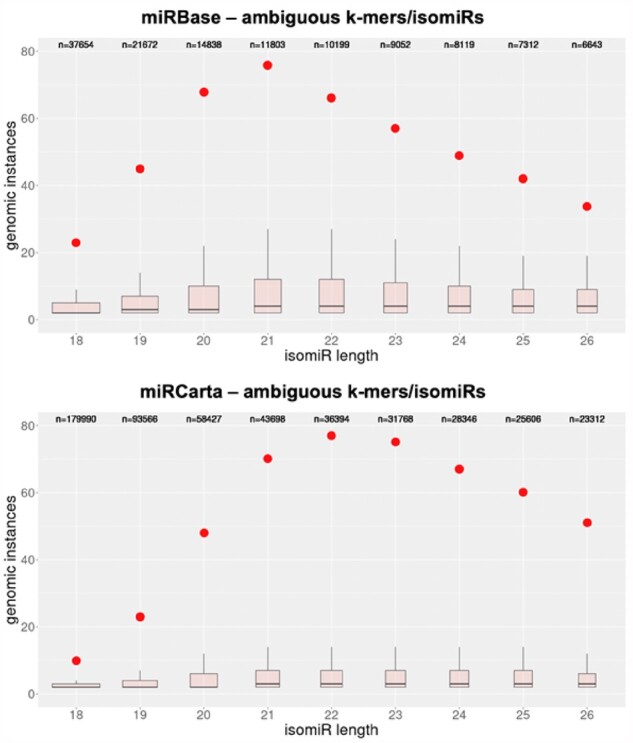
Boxplots showing the number of genomic instances for k-mers of different lengths. These k-mers represent all possible wild-type isomiRs that can be formed by the miRNA precursors contained in miRBase (top) and miRCarta (bottom), respectively, and have lengths between 18 and 26 nts. In each panel, the red dots represent the value at the 90^th^ percentile of the corresponding boxplot. Note that the number of distinct k-mers (shown above each boxplot as “n=xxxxx”) differs considerably for different values of k

For miRBase, 27.4% of the isomiRs with length exactly equal to 18 nts are ambiguous. The portion of ambiguous isomiRs continues to remain high (10.8%) when isomiRs with lengths >18 nts are considered. For the miRCarta reference set, the percentages of ambiguous isomiRs are 22.3% and 7.7%, respectively. These statistics prompted us to examine the sequences of all possible isomiRs that can be formed from the known miRNA precursors and have lengths between 18 and 26 nts inclusive. These isomiRs in fact correspond to the k-mers of the k-mer lookup table. We analyzed exclusive and ambiguous isomiRs/k-mers separately for each of miRBase and miRCarta. For all values of k between 18 and 26 nts, nearly all exclusive isomiRs have one genomic instance (data not shown). Figure 3 shows the boxplots for the ambiguous isomiRs/k-mers of miRBase and miRCarta. It is evident that for all values of k both databases contain k-mers, i.e. potential isomiRs, which can be found at many genomic locations. Considering these observations, isoMiRmap’s ability to identify ambiguous isomiRs and tag them as such provides users with valuable information.

### 3.4 Automated identification of isomiRs that correspond to conflicting annotations or previously unannotated miRNA arms

It is also possible that a given deep sequencing dataset supports the existence of isoforms that extend beyond the boundaries of the nominal precursor sequence found in the public miRNA databases. For example, several precursors that are listed in miRCarta are truncated instances of the precursors that are listed in miRBase, likely due to their re-annotation process (Backes *et al.*, 2018). Examples include hsa-119-538.1/hsa-mir-34a, hsa-2636.1/hsa-mir-4251 and hsa-207.1/hsa-mir-429. In such cases, an isomiR would seem to extend beyond the confines of a miRNA precursor, which is not the case. Clearly, provisions need to be made to enable the reporting of isomiRs independent of which of the two databases users select for their analyses.

Moreover, abundant isomiRs can arise from miRNA precursor arms previously thought to be ‘silent.’ Two characteristic examples include the 5p arms of hsa-mir-103 and hsa-mir-107. Figure 6 of Londin *et al.* (2015) shows several distinct and very abundant isomiRs being produced from the 5p arms of these two precursors. Notably, these isomiRs appear to be produced in a tissue-type specific manner (Londin *et al.*, 2015). While both of these 5p arms are included in miRCarta, as of Rel. 22 of miRBase, the 5p arm of hsa-mir-107 remains to be annotated.

IsoMiRmap is designed to accommodate the possibility that a precursor’s arm may not be annotated in the public databases as being a source of mature miRNAs. This is achieved by isoMiRmap considering the entire length of the miRNA precursor as a potential source of isomiRs. Moreover, to accommodate the possibility that the reported miRNA precursor may be a truncated version of the true one, isoMiRmap extends each precursor arm by six nts on each side (see Section 2).

### 3.5 Exhaustive reporting of overlaps with repeat elements

IsoMiRmap also reports whether a given isomiR exists within a known repeat element. As deep sequencing approaches become more popular, there is an increase in the reporting of putative miRNAs using automated procedures. On occasion, this led to mis-reporting as miRNAs short RNAs that arise from repeat elements such as tRNA (Schopman *et al.*, 2010).

Let us look again at the iso-21-W05I2PWPE isomiR of Figure 2. This is tagged by isoMiRmap as being ambiguous. Indeed, the sequence can be found in multiple LINE copies across the genome and isoMiRmap alerts the user to that effect. It is conceivable that this short RNA is transcribed independently from one of these LINE-containing loci. Repeat elements such as LINE-1 (Yang and Kazazian, 2006) and Alu (Babiarz *et al.*, 2008) are known to produce small interfering RNAs with lengths that resemble those of isomiRs. In addition, tRNA and rRNA are known to produce, in a regimented manner, short RNAs that are dubbed *tRNA-derived fragments* (Pliatsika *et al.*, 2016, 2018; Telonis *et al.*, 2015b) and *rRNA-derived fragments* (Cherlin *et al.*, 2020), respectively.

As one might expect, the ambiguous isomiRs that are contained in our k-mer table (see Section 2) are enriched in overlaps with repeat elements compared to the exclusive isomiRs. For the miRCarta reference set, 31.1% of the ambiguous isomiRs overlap repeat elements compared to 6.3% for the exclusive isomiRs, a nearly five-fold increase. For the miRBase reference set, the percentages are 35.8% and 6.5%, respectively, i.e. miRBase’s ambiguous isomiRs show an enrichment in repeat element overlap as well.

While it may be counterintuitive, it is nonetheless important to stress the observation above that *exclusive* isomiRs can overlap one or more RepeatMasker classes. This can happen with parental miRNA precursors that entered the literature without having been filtered using the most up-to-date repeat elements annotations. While these isomiRs could be arising from double-stranded, hairpin-shaped precursors, they may belong to other categories of short interfering RNAs instead of being *bona fide* miRNAs. In the event that an isomiR overlaps multiple repeat element classes, isoMiRmap will report the name of each such class in the form of a comma-separated list.

### 3.6 Support for 3′ non-templated additions

The post-transcriptional addition of non-templated nucleotides to the 3′ ends of mature miRNAs is a process whose details are not well understood. The non-templated additions can involve a single or multiple nucleotides (Song *et al.*, 2015). Any one of the four nucleotides can be added post-transcriptionally, albeit with different frequencies (Yang *et al.*, 2019a). The modification could take place at the level of pre-miRNA as a mechanism to control miRNA production (Newman and Hammond, 2010) and propagated to the production of mature miRNAs from the 3p arm of miRNA that inherit these non-templated additions (Kim *et al.*, 2019). These modifications were shown to have diverse roles including modulation of miRNA target effectiveness (Burroughs *et al.*, 2010; Yang *et al.*, 2019a), and stabilization (Katoh *et al.*, 2009). Of note, 3′-end uridylated isoforms have also been reported in exosomes (Koppers-Lalic *et al.*, 2014).

IsoMiRmap can identify and can guarantee the reporting of isomiRs whose 3′ non-templated additions include one *or more* instances of either A, C, G or U. IsoMiRmap can report isomiRs with potentially long stretches of 3′ additions. This is because the maximum length constraint (26 nts) is enforced only on the *templated* portion of the isomiR.

Any discovered isomiRs with 3′ non-templated additions are included in the same output files as the templated isomiRs and are accompanied by similar metadata. The ‘Type’ column for these non-templated molecules indicates the type and length of the additions. For those entries, the source hairpin(s), alternative labels and miRGFF3 (Desvignes *et al.*, 2020) output are updated accordingly in order to capture the event.

### 3.7 Seamless reporting of mutation-containing and polymorphism-containing isomiRs

IsoMiRmap also reports instances of isomiRs that contain somatic mutations or common polymorphisms compared to the reference genomic sequence. It achieves this by incorporating the relevant information in a second k-mer lookup table (see Section 2). By introducing a second table, isoMiRmap can generate additional information without affecting performance. The reporting of variant-containing isomiRs includes the following information: isomiR sequence containing the variant, templated sequence, unnormalized and normalized abundances, parental precursor and the corresponding license plate. In addition, isoMiRmap reports the applicable dbSNP, gnomAD or COSMIC identifier(s). In total, 19071 unique variants are profiled (6152 common SNPs and 12919 somatic mutations). In the case where the variant-containing isomiR sequence can be generated by more than one SNP identifier and precursor combinations, all are listed. Variant-containing isomiRs are reported separately from the wild-type isomiRs. No RepeatMasker overlaps or miRNA-space exclusivity information are reported for variant-containing isomiRs.

**Fig. 4.**
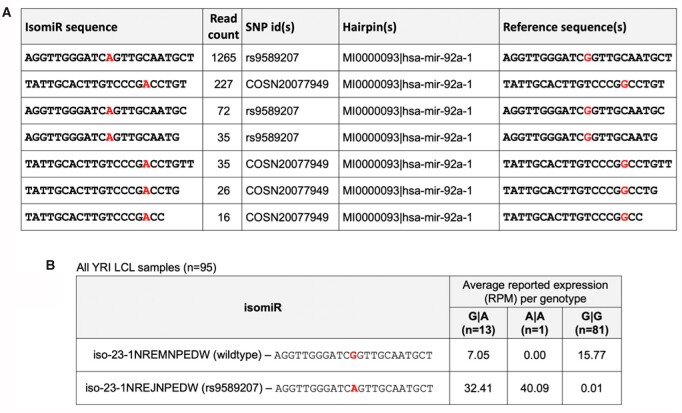
Variant-containing isomiRs. (**A**) A subset of isoMiRmap’s output from the analysis of sample NA19257 (YRI population from the 1000 Genomes Project). Only SNP-containing isomiRs from hairpin hsa-mir-92a-1 with support by ≥10 reads are shown. Normalized read counts, hairpin coordinates, the molecules’ license plate and the unique identifier for the reference miRNA sequence were excluded for clarity. (**B**) Expression values across all samples from the YRI population (*n* = 95). IsoMiRmap correctly identifies all three (G|A, A|A, G|G) genotypes across the samples

We illustrate the value of isoMiRmap’s SNP calling with the help of sample NA19257 from the 1000 Genomes Project (1KG) (Genomes Project Consortium *et al.*, 2015; Lappalainen *et al.*, 2013). This sample belongs to a donor from the YRI (Yoruba in Ibadan, Nigeria) population group. The most abundant variant-containing isomiR from this sample arises from the miR-92a-1 locus of the miR-17/92 cluster (Mogilyansky and Rigoutsos, 2013). Figure 4 shows isoMiRmap’s output for the variant-containing isomiRs from this precursor that are supported by ≥10 reads. These isomiRs contain a somatic mutation (COSN20077949) and a germline variant (rs9589207), each at different locations. Both variants correspond to a G→A substitution.

We also investigated the potential presence of rs9589207 in the remaining 94 samples from the YRI population for which short RNA and DNA data are available. As can be seen in Figure 4B and Supplementary Table S1, isoMiRmap correctly identifies all three (G|A, A|A, G|G) genotypes across the samples. We found no significant expression of iso-23-1NREJNPEDW in the other four population groups, namely CEU (Utah Residents with Northern and Western European Ancestry), FIN (Finnish in Finland), GBR (British in England and Scotland) and TSI (Toscani in Italia).

### 3.8 Exhaustive and deterministic identification and quantification of isomiRs

In keeping with the approach that we introduced in MINTmap (Loher *et al.*, 2017), isoMiRmap guarantees that it will produce results that are both deterministic and exhaustive, even though it does not need to access a copy of the genome assembly during run-time. The deterministic and exhaustive nature characterize all of the tool’s stages, including: when identifying hairpin locations, when identifying SNP variants, when finding mature intersections, and when determining whether an isomiR is exclusive or ambiguous. To prevent the occurrence of false negatives and to be able to comprehensively report all genomic instances of an isomiR, isoMiRmap obviates the use of probabilistic schemes, or measures that evaluate the goodness of an alignment. The use of the k-mer table has an additional benefit. Namely, it makes all of the processing be ‘isomiR-oriented’ (and not genomic-location-oriented). This means that isoMiRmap determines isomiR abundances separately for each distinct isomiR sequence independently of how many genomic instances the sequence may have. In doing so isoMiRmap avoids double-counting, a consideration that is particularly relevant for isomiRs with multiple genomic copies (see Fig. 3).

### 3.9 High-performance with minimal resource and user requirements

As mentioned above, isoMiRmap carries out its searches by relying on the use of pre-computed lookup tables (see Section 2). Because the tables are modest in size, isoMiRmap can be run on a standard laptop and still offer exceptionally high performance. For example, using miRBase as the underlying reference database, isoMiRmap will process 10 million sequenced reads in under 55 s on an Intel Core i7 2.90 GHz processor. We also tested isoMiRmap with different inputs and confirmed that the runtime scales linearly with the size of the input, as would be expected given that the algorithm is hash-table based. E.g., isoMiRmap will process 30 million sequenced reads in approximately 125 s. See Supplementary Figure S2 for more information. In the same amount of time, in addition to profiling isomiRs, isoMiRmap will report exclusivity/ambiguity information, enumerate all overlaps with repeat elements, and report any polymorphisms that may be present.

IsoMiRmap is easy to install and run: it is a self-contained, plug-and-play tool that does not require the installation of any third-party mapping tools or, a local copy of the genome. IsoMiRmap is built with Python (Version 3+) and, thus, can run on all major operating systems including Linux, OS X and Microsoft Windows. The user need only provide a file that contains the sequenced reads, after quality trimming and adapter removal. This file should be in the FASTQ format and can be uncompressed (e.g. fastq) or compressed (e.g. fastq.gz). A built-in help file provides access to this information.

The output style of isoMiRmap is very similar to that of MINTmap (Loher *et al.*, 2017). The isomiR profiles are generated in three formats: tab-separated text files, HTML and miRGFF3. The text files are meant to facilitate further analyses using software such as Excel, R or Matlab. The HTML output provides an overview through all major web browsers. Importantly, in the output files, isoMiRmap reports information separately for each isomiR sequence. This can prevent multi-counting in those instances where a given isomiR can arise from multiple precursors. Lastly, we generate output in the recently introduced miRGFF3 (Desvignes *et al.*, 2020) format to facilitate conversion into other formats and enable the incorporation of isoMiRmap in existing analytical pipelines. In addition to reporting the discovered isomiRs, the output includes summary statistics including the total number and percentage of reads that map exclusively, ambiguously or include genomic variants.

### 3.10 Comparisons with similar tools

In this section, we evaluate isoMiRmap and four other tools, SeqBuster V3.2 (Pantano *et al.*, 2010), the BCGSC miRNA profiling pipeline (Chu *et al.*, 2016), miRquant V2.0 (Kanke *et al.*, 2016) and miRge V2.0 (Lu *et al.*, 2018).

Of the five tools, isoMiRmap, BCGSC and miRquant take into account the entire genome assembly when processing the sequenced reads. The remaining tools, SeqBuster and miRge, consider only the sequences contained in a reference miRNA database. In our comparisons, we considered only isomiRs with lengths between 18 and 25 nts, as these lengths are supported by all five tools. We profiled isomiRs across several datasets from The Cancer Genome Atlas (TCGA) repository. Specifically, we analyzed all four short RNA normal samples from TCGA’s pancreatic ductal adenocarcinoma study (see Materials and methods). In addition, to gauge the performance of the five tools when presented with highly similar isomiRs, we designed and used three synthetic datasets: they comprise all isomiRs in the allowed length range from the precursors of the let-7 family, the mir-17/92 cluster, the latter’s paralogue clusters mir-106a/363 and mir-106b/25 (Mogilyansky and Rigoutsos, 2013), and the mir-320 family that is known to favor targets in the coding sequences of mRNAs (Wang *et al.*, 2010). We created the three synthetic datasets by including each distinct isomiR with 1, 100 and 1000 copies, respectively.

First, we describe several of the more notable differences with regard to the high-level characteristics of the various tools. For a complete list see Table 1. For example, BCGSC is the only tool that does not report how an isomiR’s endpoints differ from those of the reference miRNA. Also, isoMiRmap and BCGSC are the only tools that report the genomic coordinates of an isomiR. In terms of speed, isoMiRmap is between 4 and 80 times faster than the other four tools and is the only tool that flags isomiRs as potentially arising from outside of annotated miRNA precursors (‘ambiguous’ isomiRs). We note that some comparisons involving miRquant were not possible because it differed in two ways from the other tools. First, the latest tables that are available for it are based on the older GRCh37 (hg19) assembly instead of the more recent GRCh38 (hg38) one. And, unlike the other four tools, miRquant’s reports include data that result from aggregating the abundances of all 3′ isomiRs.

**Fig. 5.**
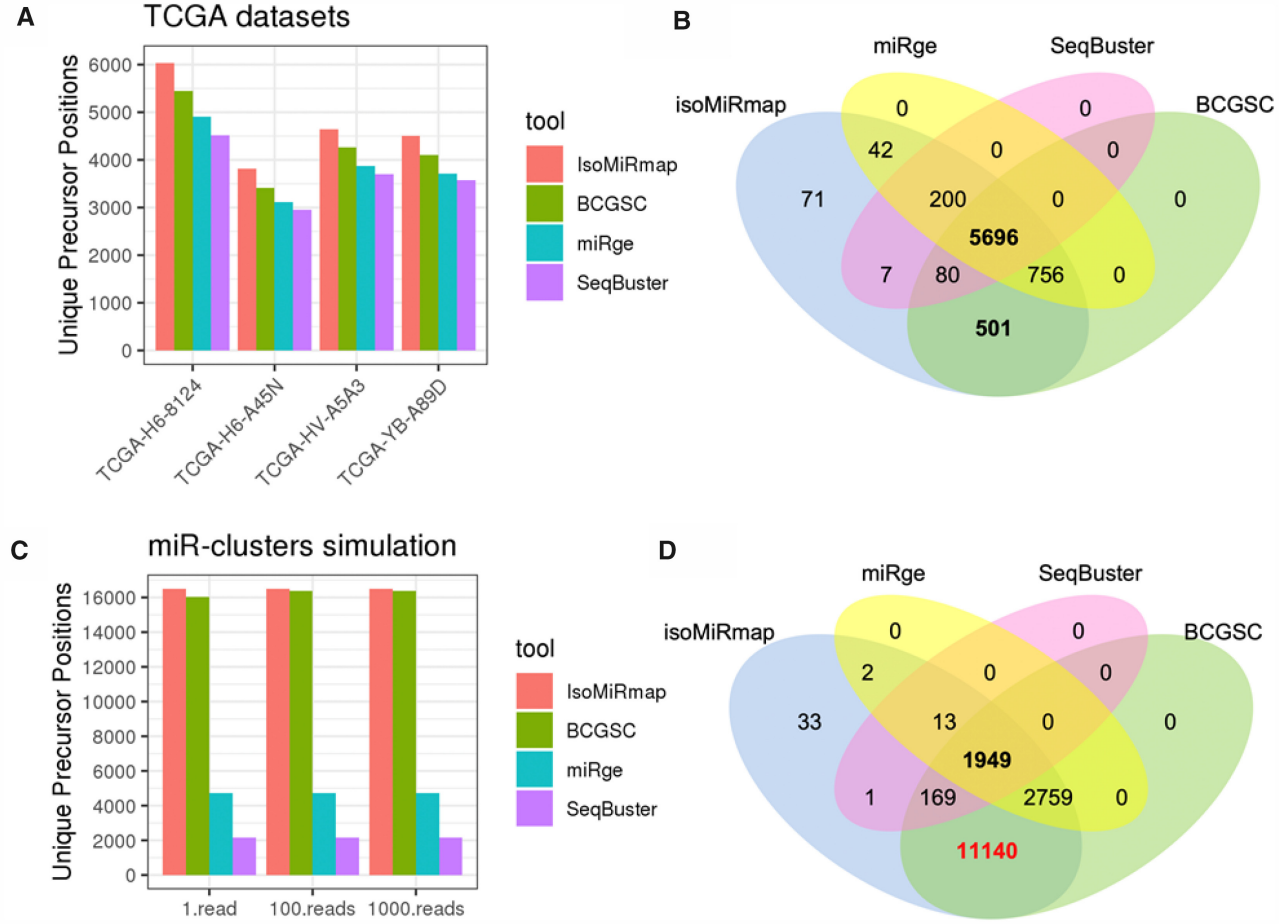
Tool comparisons. (**A**) Number of distinct precursor positions that can be the source of wild-type isomiRs that are reported by each of the studied tools. The analyzed datasets comprised four datasets from the TCGA repository (see text). (**B**) Overlap of the unique wild-type isomiR sequences that were reported by each tool for the TCGA datasets. (**C** and **D**) Similar to A and B respectively but for the three synthetic datasets comprising all possible wild-type isomiRs from the let-7 family, mir-17/92 (plus paralogues) cluster and miR-320 family (see text)

When examining the specifics of the reported results, the differences among the various tools become evident. Figure 5A shows the relative differences in the numbers of distinct precursor instances that can be sources of wild-type isomiRs and were identified by each tool in the four processed TCGA datasets. For these comparisons, we used the latest human genome assembly (GRCh38) and miRBase Rel. 22. The Venn diagram of Figure 5B shows how the four tools compare with one another in terms of the identified wild-type isomiR sequences across the analyzed TCGA datasets. As can be seen, isoMiRmap finds the highest number of wild-type isomiR sequences and parental precursor instances. Figures 5C and D show the analogous summaries for the three synthetic datasets mentioned above. Each dataset contains all wild-type isomiRs from the corresponding miRNA precursors at 1, 100 and 1000 copies, respectively.

Supplementary Table S2 contains examples of wild-type isomiR differences between the output of the different tools. An exhaustive reporting on wild-type isomiRs for the four TCGA datasets appears in Supplementary Tables S3–S5. Similarly, the outputs on the three synthetic datasets are listed in Supplementary Tables S6–S8. Inspection of these outputs allows us to make several observations. For example, miRge does not report more than one precursor for any isomiR sequence. And, BCGSC discards isomiRs if they have more than three genomic instances (e.g. iso-18-XKVLRY0I, iso-18-BJ93X2DY). It also discards the reporting of low-expression isomiRs whose expression cannot be divided among its multiple likely precursors (e.g. iso-21-BJ93X2RFB). In the case of BCGSC, we note that obtaining the expression level for an exclusive isomiR requires careful post-processing (e.g. iso-21-0317PFZ3E); in the case of ambiguous isomiRs (e.g. iso-19-B0NKZ0EX), it is not possible to do so. SeqBuster and miRge do not report isomiRs unless they overlap with an annotated mature miRNA.

### 3.11 Code availability

The isoMiRmap program and source codes are available at both https://cm.jefferson.edu/isoMiRmap/ and https://github.com/TJU-CMC-Org/isoMiRmap/ under an open source GNU GPL v3.0 license. The current implementation includes two bundles, one that uses human miRNAs from miRBase as the reference, and a second that uses human miRNAs from miRCarta.

### 3.12 IsomiR profiles for two large public repositories

We used isoMiRmap to process the 482 lymphoblastoid cell transcriptomes (Lappalainen *et al.*, 2013) of the 1000 Genomes Project (Genomes Project Consortium *et al.*, 2015), and the 10603 transcriptomes of TCGA spanning 33 cancer types. We make the expression profiles available for download in the form of compressed files at https://cm.jefferson.edu/isoMiRmap/. For ease of use, we grouped the results by population (1000 Genomes Project) or cancer type (TCGA). In each case, we make available all three output types (HTML, tab separated, miRGFF3).

## 4 Discussion

We presented isoMiRmap, a self-contained tool for profiling isomiRs in raw deep-sequencing datasets. IsoMiRmap is an open source, plug-and-play tool with minimal resource requirements. It does not require any third-party mappers or a local copy of the genome. As such, anyone can install and use it on her/his laptop in a matter of minutes. While the tool obviates the need for explicit mapping to the full genome, it nonetheless guarantees the deterministic and exhaustive reporting of isomiRs that may be present in the input file. IsoMiRmap achieves this through the use of precomputed lookup tables that can accommodate the profiling of human miRNA isoforms from two collections of miRNA precursors: those contained in miRBase and those contained in miRCarta.

It is important to note that isoMiRmap is genome-aware by design. During a computationally intensive preprocessing stage, which occurs as part of creating the tool's tables, we combine sequence information from the collection of miRNA precursors with existing genome-wide annotations. At the end of this stage, we have generated the needed tables that are key to achieving speed during mapping. Clearly, the miRNA precursor sequence information can change from organism to organism (Londin *et al.*, 2015), and species to species (Johnston and Hobert, 2003; Lin *et al.*, 2007; Stark *et al.*, 2007). Genome-wide annotations have analogous dependencies as well. Consequently, a given instance of isoMiRmap (e.g., the one designed for the human genome) cannot be used to analyze deep sequencing data from e.g. another primate. Nonetheless, the isoMiRmap methodology is directly applicable to any organism of one’s choosing: starting with a list of the miRNA precursors for this organism as well as a genome-wide list of annotations, one can follow the steps we outlined in Section 2 to create the needed tables and build the instance of isoMiRmap. These tables can be incorporated in a plug-and-play fashion to derive instances of isoMiRmap for additional genomes, and we currently plan to do so for other organisms. It is important to emphasize that isoMiRmap is not a ‘discovery tool’ but rather a mapping/annotation tool that has taken into account all recent research and can identify all isomiRs produced by a miRNA arm including those with 3′ non-templated additions and mutations. As such, it requires a reference database of miRNAs and a reference database of mutations. IsoMiRmap currently links to two reference databases of miRNAs, miRBase and miRCarta, and three databases of polymorphisms/mutations, dbSNP, gnomAD and COSMIC. Should new releases of these databases become available, ‘updating’ isoMiRmap is tantamount to computing and releasing new tables for isoMiRmap's users to download and use locally.

IsoMiRmap reports the identified isomiRs using three output formats. The tab-separated text output is meant for subsequent use by spreadsheet programs, (e.g., Excel), or analytical software packages such as those found in R and Matlab. The HTML-formatted output provides an easy to access, bird-eye’s view of the results that can be inspected using any modern web browser. Finally, the miRGFF3 (Desvignes *et al.*, 2020) output can be used to convert isoMiRmap’s output to other popular formats and facilitates inclusion of the tool in existing analytical pipelines. IsoMiRmap reports the expression levels of isomiRs in terms of raw sequenced reads and in reads-per-million . We intentionally report various types of normalization to facilitate the interworking with downstream tools such as those that calculate differential expression. For example, DESeq2 (Love *et al.*, 2014), a popular differential expression tool, normally works with raw number of reads supporting a molecule, whereas t-tests would benefit from using normalized abundance values: both are reported by isoMiRmap.

To assign labels to isomiRs, isoMiRmap uses a universal labeling scheme to which we refer as the ‘license plates.’ We originally introduced the license plate approach as a candidate solution to the problem of labeling short tRNA fragments in a consistent manner and without requiring the need of a brokering mechanism (Pliatsika *et al.*, 2016, 2018). Even though it was originally applied to tRNA fragments, the labeling scheme is trivially extendable to other categories of short RNAs such as rRNA-derived fragments (Cherlin *et al.*, 2020) and isomiRs. The license plate approach has already been incorporated in the definition of the miRGFF3 format (Desvignes *et al.*, 2020). At the same time, and in recognition of the multitude of labeling schemes that are currently in use, isoMiRmap generates redundant output wherein a given isomiR is reported using each of several labeling approaches.

IsoMiRmap can also identify instances of isomiRs with one or more 3′ non-templated post-transcriptional additions of each of A, C, G or U. One, two, three or more additions will be reported if present; still, the length of the templated portion of the isomiR is limited to between 18 and 26 nts inclusive. Our analyses and recent reports indicate that such additions are frequent, diverse and occur in a cell context-specific manner.

Recent studies have revealed the importance of genomic variants present within miRNAs (Zhao, 2020). If located within the seed sequence, these variants can disrupt the targeting profiles of the miRNA. While other programs also report the presence of SNPs in isomiRs (Lu *et al.*, 2018), isoMiRmap represents the most comprehensive description of these events. While isoMiRmap does not identify *de novo* variants, it comprehensively searches, and profiles known SNPs and mutations that have been documented in dbSNP (Sherry, 2001), gnomAD (Karczewski *et al.*, 2020) or COSMIC (Forbes *et al.*, 2017) and are located within isomiRs. These variants can potentially disrupt the functional domains of an isomiR or be expressed in a manner related to a disease or population group (e.g. rs9589207; Fig. 4B).

IsoMiRmap is the fastest among several popular tools used to profile isomiRs while also outperforming the other tools in terms of the isomiRs it can identify. We demonstrated this ability using datasets from the TCGA repository as well as several synthetic inputs. We also showed that even for highly abundant isomiRs (e.g. the let-7 isomiR iso-18-XKVLRY0I) the other tools either miss completely or underreport the genomic locations from which it can arise, whereas isoMiRmap reports them exhaustively.

In closing, we reiterate four key characteristics of isoMiRmap: its speed and minimal resource requirements; its exhaustive reporting of isomiRs; its use of an ambiguity flag; and, the reporting of isomiR overlaps with repeat elements. Not only does isoMiRmap report all isomiRs that are present in a deep sequencing dataset, it also enumerates for the user all of the miRNA precursors in which the isomiR can be found. This is particularly helpful in the case of miRNA families. For isomiRs whose sequence also exist outside the boundaries of a known miRNA precursor, isoMiRmap will tag them in its output as ‘ambiguous;’ otherwise, it will report them as ‘exclusive’ to miRNA space. Lastly, and independently of whether an isomiR is exclusive or ambiguous, isoMiRmap will report its genomic overlap with known instances of repeat elements when such overlap occurs.

## Supplementary Material

btab016_Supplementary_Data
