# Supplementary material for: IsoMiRmap: fast, deterministic and exhaustive mining of isomiRs from short RNA-seq datasets
Source: Bioinformatics. 2021 Jan 20;37(13):1828–38. doi: 10.1093/bioinformatics/btab016 (PMC8317110; doi:10.1093/bioinformatics/btab016)
Supplement: btab016_Supplementary_Data [file btab016_supplementary_data.zip › IsoMiRmap.Supplemental Text.06January2021.docx]

| Supp. Material for  IsoMiRmap – fast, deterministic, and exhaustive mining of isomiRs from short RNA-seq datasets  Phillipe Loher, Nestoras Karathanasis, Eric Londin, Paul Bray, Venetia Pliatsika, Aristeidis G. Telonis and Isidore Rigoutsos |
| --- |

# Building the tables

The steps below outline how to create the files included in the mapping bundles that come with isoMiRmap. IsoMiRmap allows a user to select which mapping bundle to use during mapping and comes with mapping bundles based on both miRCarta Rel. 1.1 (Backes, et al., 2018) and miRBase Rel. 22 (Kozomara and Griffiths-Jones, 2014). The steps below outline how additional mapping bundles can be added.

## Exclusivity: the k-mer lookup table

Below are the steps used to generate the k-mer lookup table that is included in a mapping bundle. This file lists all wildtype isomiRs and specifies if they are exclusive or ambiguous.

1. Download the genomic coordinates for all hairpins in the (e.g. miRBase or miRCarta) reference set. Flank the hairpin coordinates by 6nt on both the 5p and 3p end. Note: the flanking width can be changed if needed.
2. Convert the coordinates into FASTA sequences. To allow the end-user to know the genomic location of the hairpins, make sure the FASTA header contains not only the hairpin names, but also the genomic location of each sequence (e.g. >hsa-312-93.1&WithFlank&1|+|1167118|1167188). This FASTA file is also included in the mapping bundle defined by the *HAIRPINSEQUENCES* attribute.
3. Create a list of all possible 18-26nt (inclusive) unique substrings that can be formed from each of these hairpin sequences. This list will comprise the first column of the exclusivity table and represents all possible wildtype isomiR sequences that isoMiRmap can profile. Note: the 18-26nt isomiR size range can be changed if needed.
4. Individually for each possible isomiR sequence above, mark the isomiR as ambiguous if it resides (or partially resides) in the assembly (both strands) outside of the coordinates used in step #3 above (Sup. Figure 1). Otherwise, mark the isomiR as exclusive. An exhaustive search should be used to not miss any overlaps. This information comprises the second column in the tab-separated lookup table in which a ‘Y’ indicates that the isomiR is exclusive and an ‘N’ indicates that the isomiR is ambiguous. This lookup table is included in the mapping bundle defined by the *FRAGMENTS* attribute. Below is an example row in the exclusivity table for iso-21-2BSE98ZZ0 (MIMAT0032115&hsa-miR-548ae-5p&offsets|-1|0) which marks the sequence as being ambiguous:

CAAAAGTAATTGTGGTTTTTG N

## Attributes of k-mers: overlap with known repeat elements

This section outlines steps used to create the RepeatMasker table which is included in the mapping bundle defined by the *OTHERANNOTATIONS* attribute. Both of isoMiRmap’s included mapping bundles use the *H. Sapiens* annotations from RepeatMasker (Smit, 2013-2015 <http://www.repeatmasker.org>). We downloaded the RepeatMasker coordinates directly from UCSC’s data retrieval tool(Karolchik, et al., 2004) which includes the following 20 RepeatMasker classes: SINE, SINE?, LINE, RC, RC?, RNA, scRNA, srpRNA, rRNA, tRNA, DNA, DNA?, snRNA, Retroposon, Unknown, LTR, LTR?, Satellite, Low_complexity, and Simple_repeat. The procedure for creating this table is as follows:

Separately for each RepeatMasker class:

1. Download the coordinates of all RepeatMasker locations for that class.
2. Create class islands by combining any coordinates that overlap by at least 1nt.
3. Convert these class island coordinates into their genomic sequences.
4. For each isomiR (exclusive or ambiguous) listed in the exclusivity k-mer table, mark the isomiR as being present in the respective RepeatMasker class if its’ sequence is fully contained at least one time in any one of the sequences from the class islands. An exhaustive search should be used to not miss any overlaps.
5. Create the table listing overlaps with repeats by enumerating all isomiRs present in the exclusivity k-mer table. The sequence of the isomiR should be listed in the first column, and all RepeatMasker classes (comma separated) that it is present in should be listed in the second column. The two columns are separated by tabs. Below is an example row in the RepeatMasker table for iso-21-2BSE98ZZ0 (MIMAT0032115&hsa-miR-548ae-5p&offsets|-1|0) which marks the sequence belonging to both the DNA and LTR RepeatMasker class:

CAAAAGTAATTGTGGTTTTTG DNA,LTR

## Accommodating variant-containing isomiRs with a second k-mer lookup table

IsoMiRmap uses a SNP lookup table to allow for the efficient capturing of common genetic variation as well as other mutation events. We create and include this lookup table for both mapping bundles. Importantly, the methods below can be used to add SNPs of interest from other databases and different SNP threshold settings (e.g. minor allele frequencies or maximum INDEL sizes), as desired. We followed the following approach when generating this table:

1. Common genetic variants were obtained from dbSNP (Sherry, et al., 2001) version 151 and the Genome Aggregation Database (Karczewski, et al., 2019). In both cases, the variants were filtered for only those that had a minor allele frequency of ≥1% in at least one of the 26 reference populations represented in the 1000 genomes project (Genomes Project, et al., 2015). To account for potential somatic mutations, somatic mutations were obtained from the Catalog of Somatic Mutations in Cancer (COSMIC) v87(Forbes, et al., 2017). In both cases, only single nucleotide changes, or insertions or deletions of no more than one base-pair was used. Note: different thresholds for minor allele frequency or SNP/INDEL allowances can be used if desired.
2. The SNPs and mutations above were intersected with the genomic coordinates of all the hairpin sequences (including the 6nt flanking region) in the used miR-space.
3. For each variant that intersected with a hairpin, the position within the hairpin was noted, and the specific base-pair was converted to the new (variant version) base-pair. From this location, a list of all possible 18-26nt (inclusive) sequences that can be formed and encompass the altered nucleotide(s) are created.
4. The lookup table for the variants (example line below) contains the following information: the SNP containing isomiR sequence, variant ID (dbSNP or Cosmic ID), hairpin ID from which it is contained in, and the corresponding parental isomiR sequence. In the case that the same isomiR sequence can be generated from multiple hairpins or SNP events, all combinations are listed. This lookup table is included in the mapping bundle defined by the ‘*SNPS’* attribute. Below is an example row from the lookup table for an isomiR that could have originated from multiple variant IDs from two different hairpins.

Column 1: AAAAAGCTGGGTTGAGAGG

Column 2: [COSN18722068], [COSN5028503]

Column 3: [MI0003778|hsa-mir-320c-1&WithFlank|18|+|21683512|21683595], [MI0008192|hsa-mir-320d-2&WithFlank|X|-|140926154|140926237]

Column 4: [GAAAAGCTGGGTTGAGAGG], [GAAAAGCTGGGTTGAGAGG]

**Supp. Figure S1.** An outline of the logic used to implement the isomiR exclusivity detection used by isoMiRmap.


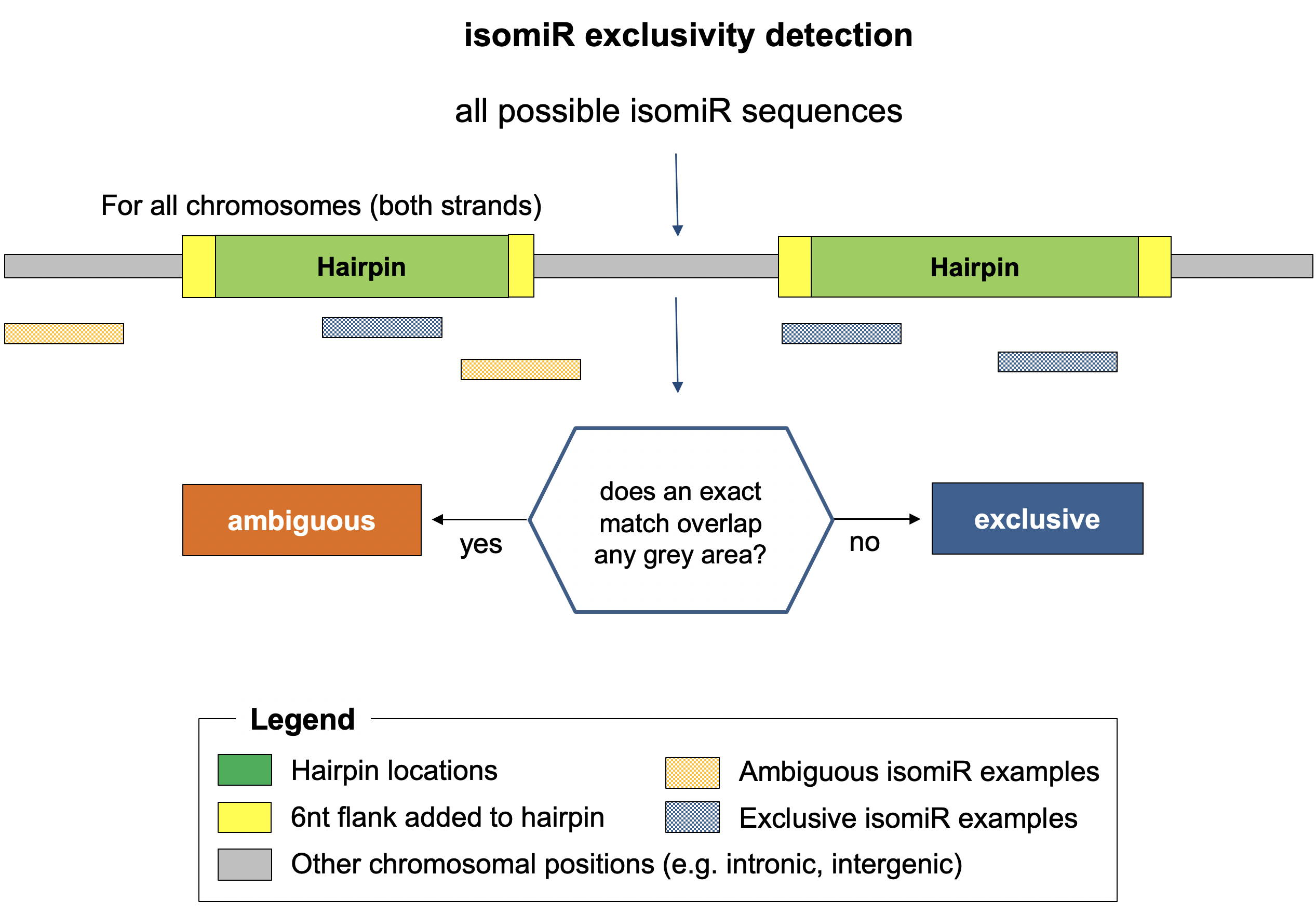


**Supp. Figure S2.** IsoMiRmap run times for inputs of increasing sizes. Note how the runtime scales linearly with the size of the input. All runs were completed on a laptop equipped with an Intel Core i7 processor running at 2.90 GHz.


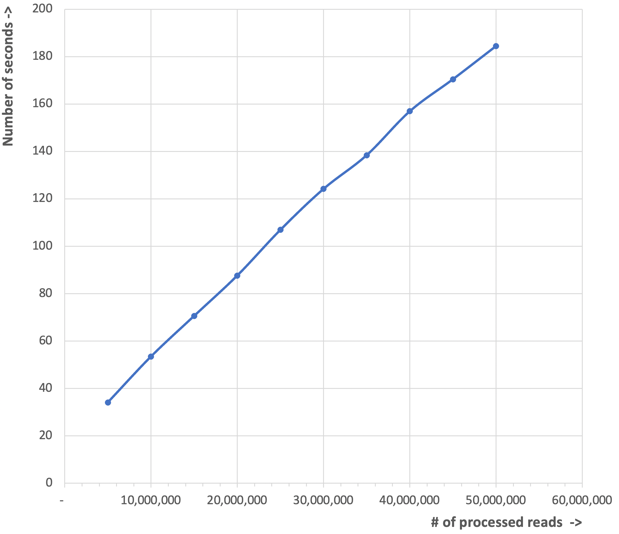


References

Backes, C.*, et al.* miRCarta: a central repository for collecting miRNA candidates. *Nucleic Acids Res* 2018;46(D1):D160-D167.

Forbes, S.A.*, et al.* COSMIC: somatic cancer genetics at high-resolution. *Nucleic Acids Res* 2017;45(D1):D777-D783.

Genomes Project, C.*, et al.* A global reference for human genetic variation. *Nature* 2015;526(7571):68-74.

Karczewski, K.J.*, et al.* Variation across 141,456 human exomes and genomes reveals the spectrum of loss-of-function intolerance across human protein-coding genes. *BioRxiv* 2019:531210.

Karolchik, D.*, et al.* The UCSC Table Browser data retrieval tool. *Nucleic Acids Res* 2004;32(Database issue):D493-496.

Kozomara, A. and Griffiths-Jones, S. miRBase: annotating high confidence microRNAs using deep sequencing data. *Nucleic Acids Res* 2014;42(Database issue):D68-73.

Sherry, S.T.*, et al.* dbSNP: the NCBI database of genetic variation. *Nucleic Acids Res* 2001;29(1):308-311.

Smit, A., Hubley, R & Green, P. . *RepeatMasker Open-4.0* 2013-2015 <<http://www.repeatmasker.org>>.
